# How we do it: the Zurich Microsurgery Lab technique for placenta preparation

**DOI:** 10.1007/s00701-023-05847-5

**Published:** 2023-11-23

**Authors:** Lara Maria Höbner, Victor E. Staartjes, Elisa Colombo, Martina Sebök, Luca Regli, Giuseppe Esposito

**Affiliations:** Zurich Microsurgery Lab, Department of Neurosurgery, Clinical Neuroscience Center, University Hospital Zurich, University of Zurich, Frauenklinikstrasse 10, 8091 Zurich, Switzerland

**Keywords:** Microsurgical training, Microsurgery, Microvascular surgery, Microsuturing, Microvascular anastomosis, Placenta, Microanastomosis, Education

## Abstract

**Background:**

Perfused placentas provide an excellent and accessible model for microvascular dissection, microsuturing and microanastomosis training — particularly in the early microsurgical learning curve. This way, a significant amount of live animals can be spared.

**Method:**

We present the Zurich Microsurgery Lab protocol, detailing steps for obtaining, selecting, cleaning, flushing, cannulating, and preserving human placentas — as well as microsurgical training examples — in a tried-and-true, safe, cost-effective, and high-yield fashion.

**Conclusion:**

Our technique enables highly realistic microsurgical training (microdissection, microvascular repair, microanastomosis) based on readily available materials. Proper handling, preparation, and preservation of the perfused placenta models is key.

**Supplementary Information:**

The online version contains supplementary material available at 10.1007/s00701-023-05847-5.

## Relevant surgical anatomy

**Fig. 1:**
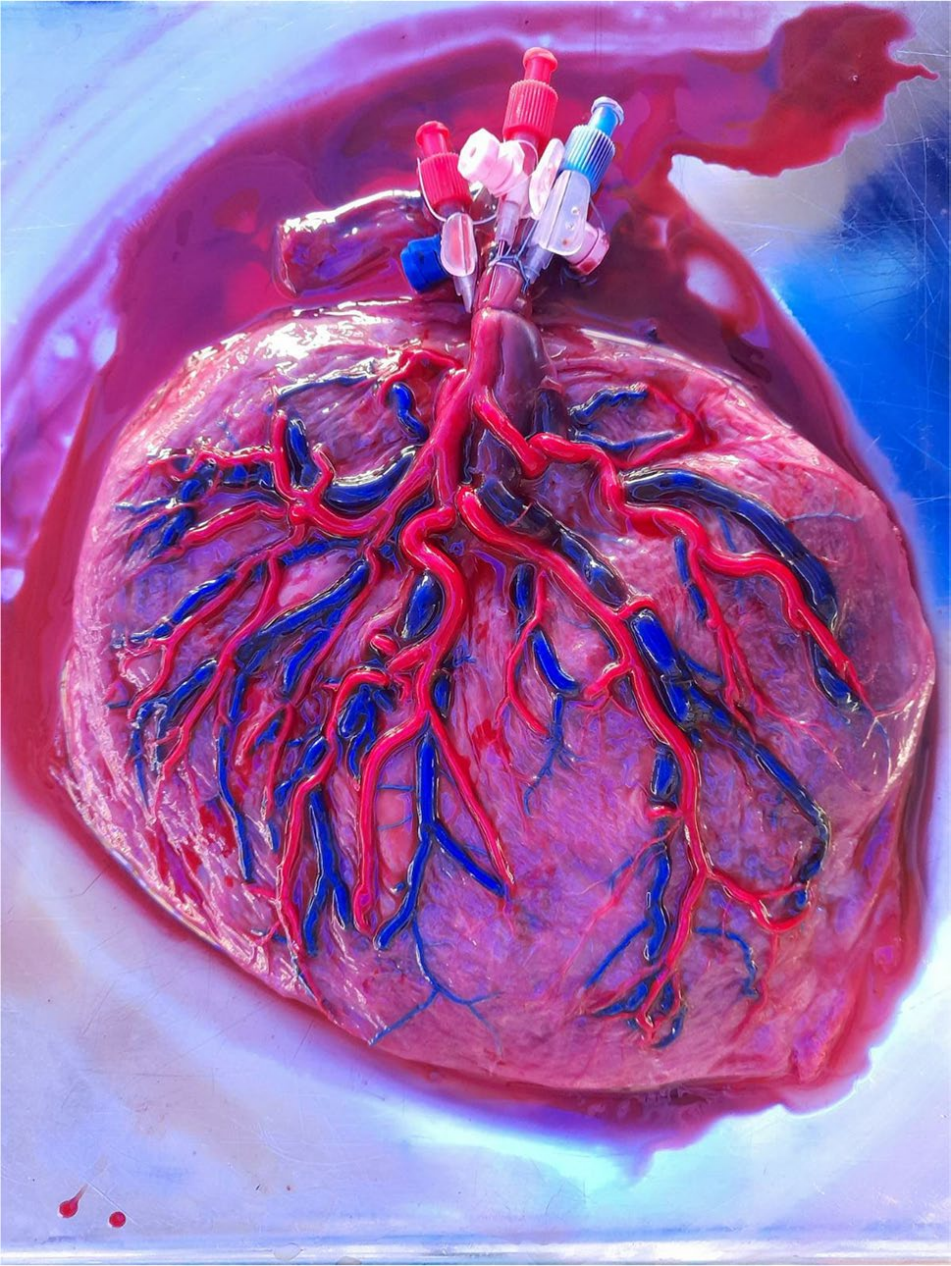
Acryl-injected placental arterial (red) and venous (blue) vascularization. Note the anastomosis of the two major arteries at the level of the umbilical cord, which is present in almost all placentas, as well as the strict crossing of arteries over veins

McGregor et al. [3] provide an excellent overview of placental anatomy for the purpose of microsurgical training. Briefly, vessels vary in size between 3–5 mm at the hilum of the cord and 0.5–2 mm peripherally. Two highly anastomosed arteries divide 3 times and strictly pass over veins. The adventitial layer, mimicking arachnoid but slightly thicker, is well connected with the stroma surrounding it, and adventitia of arteries and veins at their cross-points appear to fuse at times. The media of placental arteries is thinner and contains less elastic fibers than comparable mature vessels. Veins tend to be blood-filled and of larger diameter. The umbilical cord contains 2 arteries and 1 vein. An example of typical placental vascular anatomy is provided in Fig. 1.

## Description of the technique

The entire technique is presented in a three-minute video (Supplementary Material). The minimum selection of tools necessary for placental preparation is: 0.9% saline (or Ringer’s lactate) solution, an ampule of Heparin (5000 I.E./mL), three winged indwelling venous cannulas (preferably 2 × 22 gauge for the arteries and 1 × 20 gauge for the vein), a syringe, a blunt fill needle, surgical scissors, 2-0 ligatures, and three Luer-lock caps.

The placenta is obtained from the Obstetrics Department of the University Hospital Zurich, Switzerland and the mother signed an informed written consent form beforehand.

**Fig. 2:**
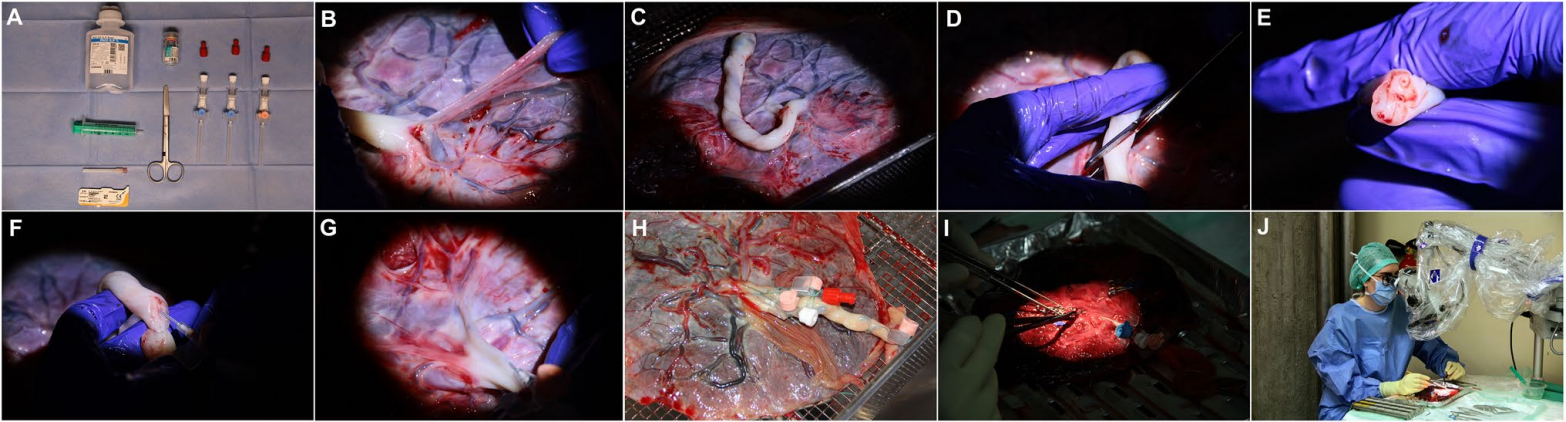
Illustration of the Zurich Microsurgery laboratory process for preparation of perfused placenta models. **A**. The minimum selection of tools necessary for placental preparation. **B**. The placenta is washed and the parietal part of the amniotic sac is cut away. **C**. Aspect of a cleaned and peeled placenta. **D**. The umbilical cord is cut obliquely. **E**. Inspection of the cutting plane reveals the two arteries laterally and the central vein with a large lumen. **F**. Venous cannulation with a large-bore catheters can be easily achieved at the umbilical cord. **G**. The arteries are best punctured at the base of the umbilical cord. **H**. Fixation techniques. **I**. Flush with heparinized saline. **J**. Placenta model in use for microsurgical training

The placenta is washed and the parietal part of the amniotic sac is removed. The visceral part of the amniotic sac (pictured here in Fig. 2 as the layer gripped in between the gloved fingers) can be peeled away from the placental surface to expose the vessels, but can also be (partially) left in place for tougher microdissection training. Figure 2 also demonstrates an aspect of a cleaned and peeled placenta. The vessels ought to be inspected: If there are only hypoplastic and extremely small-calibre vessels or if all vessels are thrombosed, placentas should be sorted out as they are usually not well-suited for microsurgical training. At least one vein and one large artery proximally (close to the umbilical cord insertion) should be clearly seen.

The umbilical cord is cut obliquely while stretched longitudinally to maximize the diameter of exposed vessel lumina. Inspection of the cutting plane reveals the two arteries laterally and the central vein with a large lumen. Although one can attempt to directly cannulate all three vessels including the arteries here, we only recommend cannulating the vein, since cannulating arteries with this technique leads to a high rate of dissection due to tortuosity. In contrast, venous cannulation with a larger-bore catheter can be easily achieved at the umbilical cord. The arteries are best punctured at the base of the umbilical cord where they are both clearly identifiable and still within the thick fibrous coverings of the umbilical cord, allowing proper fixation. While gently pulling on the cord, the artery can be cannulated directly. Note that due to the high rate of high-volume proximal anastomoses existing between the two placental arteries, proximal cannulation of one of these arteries can sometimes be enough to adequately perfuse both vessels. If not, two separate cannulas are inserted. Using 2-0 to 4-0 ligatures, the winged cannulas ought to be very tightly fixated around the fibrous umbilical cord. Sometimes, sling constructions around the base of the umbilical cord — as pictured in Fig. 2 — help to avoid pullout of the catheters during transport and training.

Once proper access has been obtained, all vessels are flushed with heparinized saline. In some cases, with lengthy thrombosis, high-dose heparin can be left within the vessels for 20 min to help dissolve or loosen the clots, which can sometimes be flushed away with moderately high pressure afterwards.

The finished models allow microdissection, microvascular repair, and microvascular anastomoses on highly realistic vessels for up to 48 h of use with intermittent cooling. Make sure to keep the placental surface and especially the vessels wet at all times.

If not used immediately, the placenta can be wrapped airtight in three layers of packaging without trapped air and can then be stored frozen at −80° C (−112° Fahrenheit) for up to 3 months in a monitored freezer. To unfreeze and prepare placentas for microsurgical training, place the firmly frozen placentas in a fridge at 4 to 7° C (39 to 45° Fahrenheit) for 24–36 h, where the tissue can slowly accommodate to the new temperature, before running under tepid water right before training use.

## Indications

Adequate training in microsurgical techniques is essential in many surgical disciplines such as neurosurgery, plastic surgery, hand surgery, maxillo-facial surgery, transplant surgery, and some veterinary surgery. Training the required microsurgical skills during clinical practice is unthinkable, as the risks for the patients are not acceptable. Therefore, teaching microsurgical skills using simulators and animals is part of the current practice. A perfused/pressurized placenta has recently emerged as a new model for microsurgical/microanastomosis training in a laboratory setting, primarily to reduce the number of live animals necessary particularly in the early microsurgical learning curve [1, 2, 4, 5]. At our institution, we have developed a standardized and, by now, established and tried-and-true protocol for preparing the perfused placenta models using tools that are readily available in any surgical department, and this model is now regularly used for microsurgical training of staff members and residents as well as during regularly held training courses.

## Limitations

While the visceral part of the amniotic sac does provide an arachnoid-like layer to practice microdissection, it is usually thicker and tougher than arachnoid membranes. This means that although this does provide for a challenge, a true simulation of Sylvian or cisternal microdissection is not entirely replaced here; however, one will find Sylvian or cisternal microdissection easier to perform, after having trained on a placenta model. Although the system can be perfused, a live animal model additionally offers the coagulative properties of blood, which is not possible in this placenta model. Lastly, while not within the realm of animal models in terms of licensing, logistics, costs, and importantly ethical concerns, these placenta models do still require the utmost hygienical care and are still resource-intensive to set up. This is, however, offset by the fact that in contrast to animal models — apart from the many other benefits of placenta models — each single placenta offers dozens of vessels of different size and consistency. In fact, on the same placenta model, the trainee can practice many iterations of microdissection, microsuturing, and all the microanastomoses types (end-to-end, end-to-side, side-to-side), as many times as needed. If one vessel is damaged during its preparation, there are many other vessels that can be used.

## How to avoid complications

- Personal protective equipment should be worn at all times.
- Work with caution when handling the placenta both during preparation and training, as every surgeon does in the operating room.
- During pregnancy, tests for common infectious diseases are carried out, and potentially infectious specimens are not selected for training, as well as specimens requiring pathological study.
- Use placentas that would otherwise have been discarded as medical waste.
- Avoid repeated freeze-thaw cycles.
- For preparation, magnification and proper lighting in form of loupes, headlights, or a microscope is recommended (but not necessary).

## Specific information for the patient

Not applicable.

### Supplementary information

ESM 1 (MP4 285591 kb)

## Data Availability

The data in support of our findings can be obtained upon reasonable request from the corresponding author.
